# Efficacy of multiple Biomarkers: NGAL, KIM1, Cystatin C and IL18 in predicting pregnancy related acute kidney injury

**DOI:** 10.12669/pjms.39.2.7494

**Published:** 2023

**Authors:** Muhammad Usman Aamir

I read the above mentioned article by Rubina Naqvi et al published in current issue of Pakistan Journal of Medical Sciences Jan-Feb. 2023 with great interest. There are some fundamental issues related not only with this article but also a lot of articles which are published mixing diagnostic tests and prognostic modelling.

I have already written one editorial about discrimination and calibration in prognostic modelling in the cardiology journal for interpretation. Firstly, for logistic regression modelling there should be at least 10 outcomes per variable even if you want to increasing the sample size by adding more control group. Secondly ROC curve analysis is done differently in both analyses. The ROC curve area in diagnostic tests is usually high as compared to prognostic model. similarly, statistician just get the ROC curves which does not make sense if goes below the no discrimination line then you have to decode the variables.

In prognostic modelling calibration is more important than discrimination. predictive value (positive and negative) cannot be assessed with the case control design, and it is strongly related with the prevalence as in the case control design and all the journals are accepting the articles with similar problems. it is really great to do such studies in Pakistan with Biomarkers. My intention is not to criticize it at all. I just want postgraduate students and junior faculty to understand clinical interpretation of these as they help a lot in decision making for patient management. I would be more than happy to contribute if any help needed
